# Two Flavonoid-Based Compounds from *Murraya paniculata *as Novel Human Carbonic Anhydrase Isozyme II Inhibitors Detected by a Resazurin Yeast-Based Assay

**DOI:** 10.4014/jmb.1910.10037

**Published:** 2019-12-30

**Authors:** Anyaporn Sangkaew, Nawara Samritsakulchai, Kamonpan Sanachai, Thanyada Rungrotmongkol, Warinthorn Chavasiri, Chulee Yompakdee

**Affiliations:** 1 Department of Microbiology, Faculty of Science, Chulalongkorn University, Bangkok 10330, Thailand; 2 Department of Chemistry, Faculty of Science, Chulalongkorn University, Bangkok 10330, Thailand; 3 Structural and Computational Biology Research Unit, Department of Biochemistry, Faculty of Science, Chulalongkorn University, Bangkok10330, Thailand; 4 Program in Bioinformatics and Computational Biology, Faculty of Science, Chulalongkorn University, Bangkok 10330, Thailand

**Keywords:** Human carbonic anhydrase isozyme II (hCAII), carbonic anhydrase inhibitor, glaucoma, *Murraya paniculata*, yeast-based assay, flavonoid

## Abstract

Human carbonic anhydrase (CA) isozyme II has been used as protein target for disorder treatment including glaucoma. Current clinically used sulfonamide-based CA inhibitors can induce side effects, and so alternatives are required. This study aimed to investigate a natural CA inhibitor from *Murraya paniculata*. The previously developed yeast-based assay was used to screen 14 compounds isolated from *M. paniculata* and identified by NMR analysis for anti-human CA isozyme II (hCAII) activity. Cytotoxicity of the compounds was also tested using the same yeast-based assay but in a different cultivation condition. Two flavonoid candidate compounds, 5, 6, 7, 8, 3’, 4’, 5’-heptamethoxyflavone (4) and 3 ,5, 7, 8, 3’, 4’, 5’-heptamethoxyflavone (9), showed potent inhibitory activity against hCAII with a minimal effective concentration of 10.8 and 21.5 μM, respectively, while they both exhibited no cytotoxic effect, even at the highest concentration tested (170 μM). The results from an in vitro esterase assay of the two candidates confirmed their hCAII inhibitory activity with IC_50_ values of 24.0 and 34.3 μM, respectively. To investigate the potential inhibition mechanism of compound 4, in silico molecular docking was performed using the FlexX and SwissDock software. This revealed that compound 4 coordinated with the Zn^2+^ ion in the hCAII active site through its methoxy oxygen at a distance of 1.60 Å (FlexX) or 2.29 Å (SwissDock). The interaction energy of compound 4 with hCAII was -13.36 kcal/mol. Thus, compound 4 is a potent novel flavonoid-based hCAII inhibitor and may be useful for further anti-CAII design and development.

## Introduction

Glaucoma is a domestic and global health care challenge that affects approximately 70 million people around the world [1]. It is a multi-factorial, complex eye disease with specific characteristics, such as optic nerve damage and visual field loss. The cause of glaucoma generally is a failure of the eye to maintain an appropriate balance between the amount of internal (intraocular) fluid produced and the amount that drains away. If the glaucoma is not treated, central vision will be decreased and then lost. However, glaucoma can be managed if it is detected early, and with medical and/or surgical treatment most people with glaucoma will not lose their sight [2-4].

In healthy people, the pressure inside the eye, the intraocular pressure (IOP), is in a steady state where the rate of aqueous inflow is equal to the rate of aqueous outflow. However in glaucoma patients, the aqueous inflow has a greater rate than outflow causing a subsequent increase in the IOP [4]. Currently, decreasing the IOP is the main approach for reducing disease progression. Eye drops are often the first choice for patient treatment, where one of the major classes of drugs approved for lowering IOP in glaucoma is carbonic anhydrase (CA) inhibitors.

All CAs (E.C. 4.2.1.1) are zinc‐containing enzymes that rapidly catalyze the interconversion of carbon dioxide (CO_2_) and water to bicarbonate and proton. This reaction is needed for many basic physiological and pathophysiological processes, such as respiration, pH and CO_2_ homeostasis, electrolyte secretion in variety of tissues and organs, bone resorption, calcification, biosynthetic reactions (such as gluconeogenesis, lipid and urea synthesis), and photosynthesis. Currently, 16 CA isozymes have been identified in mammals, and these isozymes show differences in their subcellular localization, catalytic activity, and sensitivity to different classes of CA inhibitors [5-7].

Human CA isozyme II (hCAII) is the most active of these isozymes and the major CA isozyme present in the cytosol in erythrocytes and in other tissues, including the eye [5-7]. Inhibition of hCAII in the ciliary body of the eye decreases aqueous humor secretion by inhibiting the formation of bicarbonate, which is the major anion in aqueous humor, resulting in a reduction in the IOP [6-10].

However, classical CA inhibitors, such as acetazolamide (AZA) and the topical drug brinzolamide, which have been used as commercial drugs for the treatment of glaucoma [8-10], can also inhibit other CA isoforms, diluting the drug effectiveness and causing undesired side effects from off-target inhibition. Moreover, since classical CA inhibitors are sulfonamide-based compounds and their bioisosteres, patients with a sulfa allergy cannot be treated with them. Also, rare adverse drug interactions have occurred in patients taking high doses of aspirin and CA inhibitors. Side effects of commonly used sulfonamide-based CA inhibitors cover a wide range from epidermis rash to nausea to anaphylactic shock or acute respiratory failure [11, 12]. In addition, the incidence of adverse reactions to sulfonamide-based compounds increases with age and is more commonly exhibited in women [13]. Thus, the search for safer CA inhibitors that are not sulfonamide-based is required.

Several classes of compounds, such as carboxylic acids, phenols, polyamines, coumarin and their derivatives, have been found to act as non-classical CA inhibitors [14]. All of these compounds were identified using a biochemical strategy. However, the identification of the properties of a compound in the early stage of the drug discovery process is important. A successful drug-lead candidate must possess favorable characteristics, including potency and selectivity to the biological target, minimal toxicity, good stability and physicochemical profile, and desirable absorption, distribution, metabolism, and excretion properties. However, these properties of any candidate compound cannot be obtained from drug screening using the standard biochemical strategies [15].

Drug screening should ideally be performed with cells of human origin, but they are expensive and time consuming to cultivate, while the genetic manipulation of mammalian cells is generally problematic. The yeast *Saccharomyces cerevisiae* is a simple eukaryote, but the high degree of conservation of important biological processes between yeast and human cells has made yeast a valuable, inexpensive and simple alternative system to mammalian cells for drug screening [16, 17]. The lead compounds that are identified through yeast cell-based assays have already passed important validation steps for a combination of properties that make a successful drug candidate. The availability of this information provides a head start compared to many in vitro assays and can save valuable time and costs in the development of candidate drugs [16].

A novel high-throughput yeast-based screening system for hCAII inhibitors has previously been developed [18]. The drug-sensitive *S. cerevisiae* strain lacking a gene coding for CA (Δ*nce103*) but expressing hCAII [AS03(pGAL1.1_hCAII)] was constructed and used as indicator cells. When the cells are cultivated under a low (ambient) CO_2_ condition but in the presence of galactose, the growth defect of Δ*nce103* cells is rescued by the induced hCAII expression. However, if the test compound inhibits hCAII activity in the yeast indicator cells grown under low CO_2_ condition, the growth defect is not restored. Whether the lack of yeast growth is due to a CA inhibitor activity candidate or a cytotoxic compound can be distinguished by cultivating the yeast in the presence of the test compound at a high CO_2_ condition, where in the absence of a cytotoxic affect the yeast cells can grow independent of hCAII activity. This yeast-based assay can be performed in a 96-well plate format utilizing the cell viable indicator dye resazurin [18] for ease of end result reading and so enable the assay to be used in a high-throughput screening. One of the obvious advantages of this yeast-based assay is the ability to search for safer hCAII inhibitors at the time of drug screening.

Due to their evolution of diverse defense chemicals (secondary metabolites), plants are potentially a tremendous source of diverse and valuable natural products. The disease-inhibiting capabilities of plants make them extremely useful as a source of natural drugs, and they also provide basic bioactive compounds that are less toxic, more effective, and with or without biological and chemical modification, can become potent drugs [19].

*Murraya paniculata* (L.) Jack., commonly known as Chinese box or Jasmine orange, is an ornamental and hedge plant grown for its pleasant smell and beauty as well as a source of herbal therapy for treating stomachache, toothache, rheumatism, thrombosis, cough, fever, dizzyness, colic, diarrhea, stasis of blood and body pains from injury or trauma [20]. The leaf extract of *M. paniculata* was reported to contain coumarins [21, 22] and flavonoids [23, 24], and to exert antioxidant [25, 26], anticoagulant [27], antitumor [28], antimicrobial [29-31], and anti-inflammatory activities [31, 32]. Diverse studies have reported that coumarins and flavonoids exhibit a CA inhibitor activity in vitro [33-38].

Herein, we utilized the developed yeast cell-based assay to screen for compounds isolated from *M. paniculata* for hCAII inhibitory activity and then investigated the inhibition effect of the candidate compounds. In addition, *in silico* molecular docking studies were performed to reveal how the most active CA inhibitor interacted with the hCAII enzyme.

## Materials and Methods

### Plant Material

The leaves of *M. paniculata* were collected in May 2015 at Nakhorn Nayok Province, located in central Thailand. The fresh collected samples were washed, air-dried, ground and kept until use.

### Extraction and Isolation of Compounds

Two methods for leaf extraction were performed. In the first method, powdered, air-dried leaves (5 kg) were sequentially extracted in 3 l of hexane, dichloromethane, ethyl acetate and then methanol for 7 d each at room temperature to give the crude hexane, crude dichloromethane, crude ethyl acetate, and crude methanol extracts, respectively. In the second approach, the crude methanol extract was suspended in water and extracted with hexane and ethyl acetate for three and five times, respectively. Each fraction was submitted to silica gel quick column chromatography and eluted with hexane-ethyl acetate mixture and dichloromethane-methanol mixture for the first and the second method, respectively. Similar fractions were combined after TLC examination. The compound was identified by ^1^H and ^13^C NMR recorded at 400 and 100 MHz, respectively on a Bruker Advance 400 MHz spectrometer using deuterated chloroform (CDCl_3_) and dimethyl sulfoxide (DMSO-d_6_).

### Yeast Cultivation and Compound Preparation

The yeast strain *S. cerevisiae* AS03 de (*MATa ade2-1 his3-11 leu2-3,112 trp1-1 ura3-1 can1-100 yrs1::HIS3 yrr1::trp1::* loxP *pdr1::hisG pdr3::hisG erg3::*loxP *nce103::*loxP*-URA3-*loxP) carrying plasmid pGAL1.1_hCAII [18] was used for screening compounds for hCAII inhibitor activity. The yeast was cultured in selective medium containing 1% (w/v) raffinose and without uracil and tryptophan, SR dropout uracil and tryptophan (SR-ura-trp) (0.67 g of yeast nitrogen base with ammonium sulfate without amino acids (Difco Laboratories, USA), 1 g of raffinose (Difco Laboratories, USA), 2 mg of adenine (Sigma-Aldrich, USA), 10 mg of leucine (Sigma-Aldrich) and 2 mg of histidine (Sigma-Aldrich) [18].

All tested compounds were dissolved in 100% (v/v) DMSO to a final concentration of 10 mg/ml.

### *In Vivo* Anti-hCAII Assay Using the Developed Yeast-Based Assay System

The screening for hCAII inhibitors was performed using the developed yeast-based assay with a Resazurin Microtiter Plate Assay (REMA) method for the result reading as previously described [18]. In brief, the yeast strain AS03(pGAL1.1_hCAII) was cultivated for 24 h in SR-ura-trp, diluted to 1-50 × 10^6^ cells/ml in fresh medium and an aliquot (10 μl) was added to each well of a 96-well plate, together with 80 μl of SR-ura-trp containing the respective test compound at one of three different concentrations (10-fold dilution for primary screening and two-fold dilution for determination of the MEC and MTC) of the candidate compounds in 0.5% (v/v) final concentration DMSO. After treatment with the compound for 30 min at 30°C, 10 μl of 20% (w/v) galactose was added into each well to obtain final concentration of 2% (w/v) galactose in order to induce hCAII expression. The plate was incubated at 30°C for 24 h under an ambient atmosphere (low CO_2_) with shaking to determine the MEC of the test compound. In parallel, another plate was incubated at 30°C for 24 h under 5% (v/v) CO_2_ (high CO_2_ condition) using an AnaeroPack (Mitsubishi Gas Chemical, Japan) to determine the toxicity of the candidate compound to the yeast indicator cells. A stock solution of 0.1 mg/ml resazurin sodium salt (Sigma-Aldrich) was added to each well of the 96-well plate to a final concentration of 0.03 mg/ml and incubated at 30°C in dark until the color in the wells containing only yeast cells with 0.5% (v/v) DMSO solvent changed from blue to pink, indicating the growth of yeast cells, prior to reading the result by eye.

### In Vitro Esterase Activity Assay

In vitro CA activity assay was determined according to the manufacturer’s protocol for hCAII (R&D Systems, USA) with slight modification. Candidate hCAII inhibitors were dissolved and diluted in 100% (v/v) DMSO. The compound solution was mixed with diluted enzyme solution in the wells of a 96-well plate. The 4-nitrophenyl acetate (4-NPA) (Sigma-Aldrich) solution was added to start the reaction and incubated at 25°C for 2 h, where the 4-NPA was hydrolyzed into 4-nitrophenol (4-NP) which was detected as the change in absorbance at 405 nm. The final concentration of 4-NPA and hCAII in the initial assay was 1 mM and 1 ng/μl, respectively. The enzyme inhibition was expressed as the IC_50_ (50% inhibition concentration), calculated by dose response curves with at least five concentrations. The CA inhibitor, acetazolamide (AZA) (Sigma-Aldrich) was used as a reference compound.

### Molecular Docking

The binding of compound **4** to hCAII at the atomic level was studied in silico by molecular docking using both rigid and flexible docking approaches. The crystal structure of hCAII in complex with AZA was obtained from the Protein Data Bank (PDB ID: 3HS4, Fig. 5) and used as the protein target, while AZA was defined as the center of the binding site for molecular docking using FlexX [39] and SwissDock [40]. All water molecules and AZA were removed. The atomic coordinates of AZA were directly obtained from the crystal structure (3HS4.pdb) and all missing hydrogen atoms were added by taking into account the hybridization of the covalent bonds, while the 3D structure of compound **4** was built by GaussView3.0 (Frisch MJ et al. 2004. Gaussian 03, Revision 2, Gaussian, Inc., Wallingford, CT). Each compound was then fully optimized by the HF/6–31(d) level of theory using the Gaussian09 program [41]. Both polar and non-polar hydrogen atoms were added to the protein.

The CA inhibitor, AZA was used as a reference ligand with 13 Å of sphere radius for FlexX, while a grid of 40 Å × 40 Å × 40 Å at coordinates x = -5.406, y = 3.078, and z = 15.029 in the active site was defined for SwissDock. For the molecular docking program SwissDock web service, the calculations were performed using the CHARMM force field with EADock DSS [42]. In flexible molecular docking by FlexX [39], the active site of CAII was allowed to move, while the incremental fragment placing technique was applied for ligand conformational flexibility. Atomic charges of ligand were assigned using the Gasteiger–Marsili formalism [43]. The Kollman all-atom charges and atomic solvation parameters were then assigned. Subsequently, the ligand was docked into the active site of CAII with 500 independent docking runs for FlexX, with the following parameters: WANTEDCONFS: 5000, NBFACTSEVAL: 5000, NBSEEDS: 250, SDSTEPS: 100, ABNRSTEPS: 250, CLUSTERINGRADIUS: 2.0, and MAXCLUSTERSIZE: 8 were applied for SwissDock. Results were visualized by UCSF Chimera package [44] and Accelrys Discovery Studio 2.5 (Accelrys Inc.). Note that the docking procedure was validated by re-docking of the original ligand AZA back to the enzyme active site.

### Statistical Analysis

Statistical analyses were performed by GraphPad Prism, version 5.01 (GraphPad Software Inc., USA) with one-way analysis of variance, followed by the Dunnett post-test for MEC and MTC determinations. Whereas, IC_50_ values were analyzed under dose-response inhibition (log[inhibitor] vs. response-variable slope) model. Each determination was performed in triplicate. Statistical significance was accepted at the *p* < 0.05 level.

## Results and Discussion

### Compound Identification

**Fig. 1:**
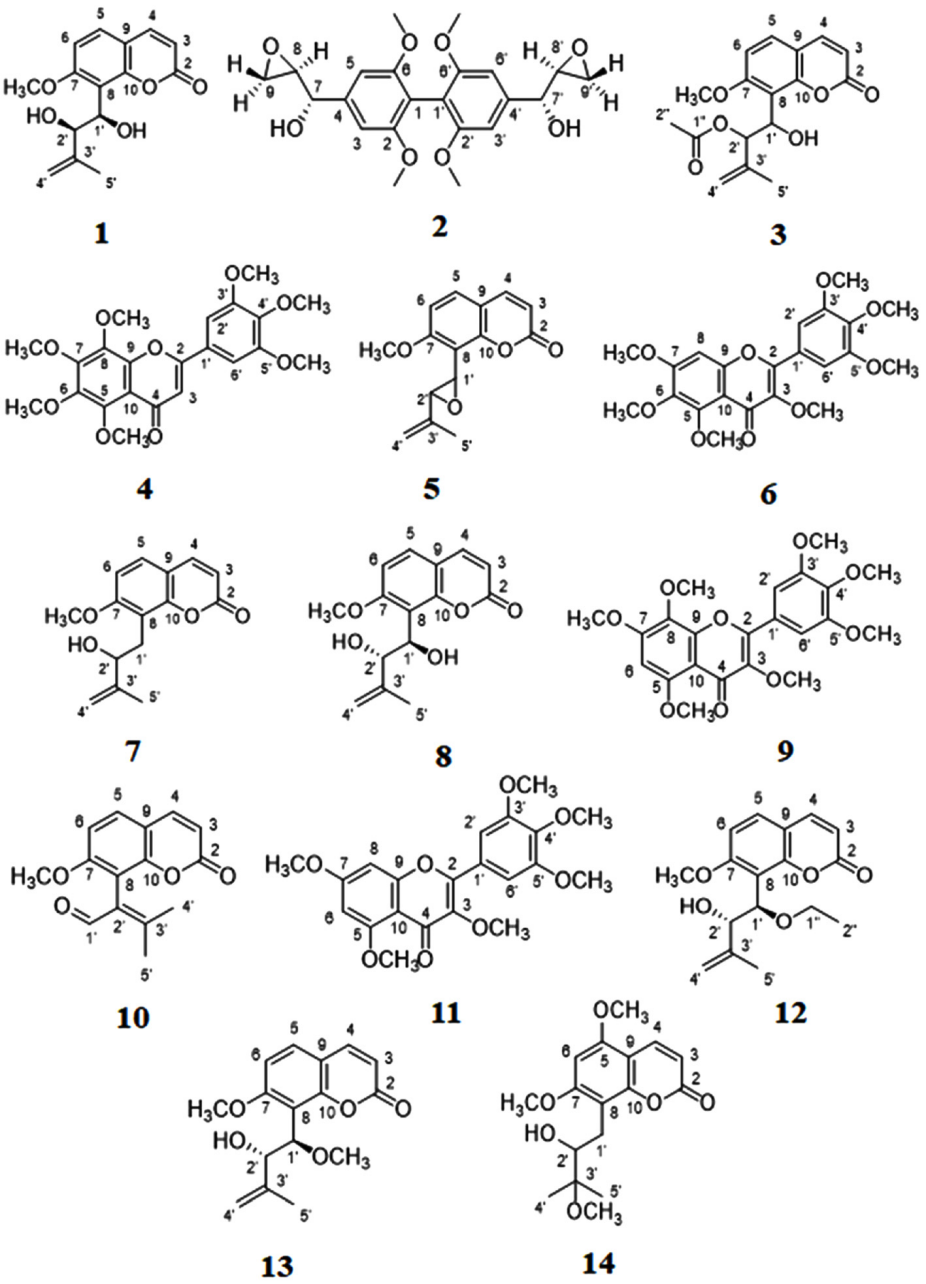
Chemical structure of test compounds.

Chemical investigation of the leaf extracts from *M. paniculata* resulted in the isolation and characterization of 14 known compounds (Table 1), identified on the basis of ^1^H- and ^13^C-NMR spectra by comparison with literature. The deduced structures of these 14 compounds are shown in Fig. 1.

### Screening for hCAII Inhibitors Using the Developed Yeast-Based Assay

**Fig. 2:**
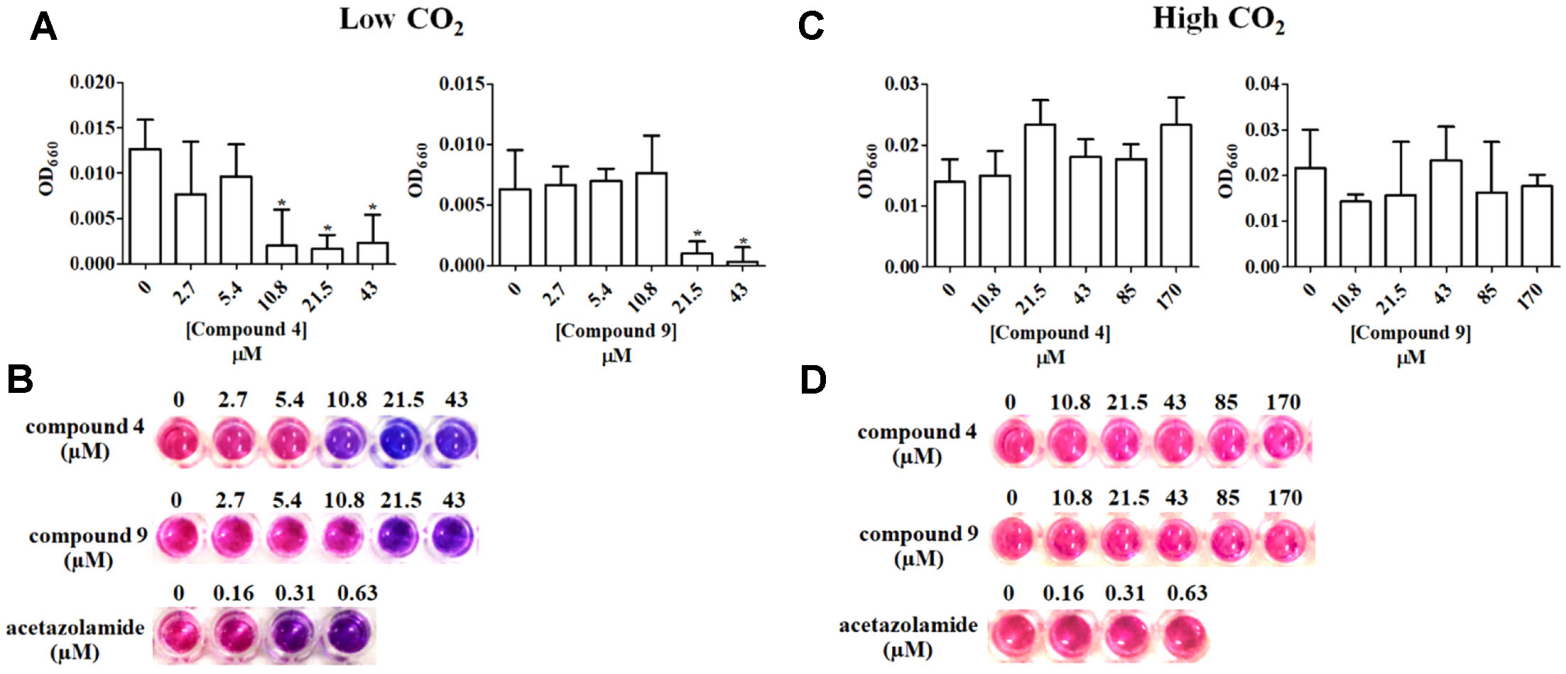
Determination of the MEC and MTC of compounds 4 and 9 against hCAII expressed in the yeast indicator cell. The growth of yeast AS03 strain carrying pGAL1.1_hCAII at different test compound concentrations under either a (**A** and **B**) low (ambient atmosphere) or (**C** and **D**) high CO_2_ (5%) condition 24 h after treatment was assessed by (**A** and **C**) measuring the OD_660_ or (**B** and **D**) the REMA method. Results are expressed as the MEC and MTC of the compound that inhibited the yeast growth under a low or high CO_2_ condition, respectively, as the (**A** and **B**) mean ± 1SD or (**C** and **D**) representative images from three independent experiments. In (**A** and **B**), statistical significance is analyzed using one-way ANOVA and Dunnett’s test, where *represents a significant difference at *p* < 0.05 compared to that of the untreated control group.

In order to investigate the compounds isolated from *M. paniculata* (Fig. 1) for the ability to inhibit the glaucoma-related hCAII activity, we first screened the 14 isolated compounds for anti-hCAII activity using the yeast cell-based assay [18]. This drug-sensitive *S. cerevisiae* strain (AS03) lacks the gene coding for CA (Δ*nce103*) but is transformed with a plasmid harboring *hCAII* under the control of *GAL1* promoter (galactose inducible). When grown under a low CO_2_ condition, growth occurs only with the galactose-induced expression of hCAII, and so can be used as an indicator strain for hCAII inhibition. When cultivated with 0.63 μM AZA, a known CA inhibitor, under low (ambient) CO_2_ condition, the yeast cells showed the growth defect with no color change in the presence of the viability indicator dye resazurin (Fig. 2B, left panel), whereas under a high CO_2_ condition, normal growth occurred with a change in the color of resazurin dye from blue to pink (Fig. 2D, right panel), indicating that AZA was not toxic to the cells and the assay was working.

To screen for hCAII inhibitors using this yeast-based assay, all 14 compounds isolated from *M. paniculata* (Fig. 1) were first separately tested at three different concentrations in 0.5% (v/v) dimethyl sulphoxide (DMSO), the highest concentration that was not toxic to the yeast indicator cells [18]. The 96-well assay plate was incubated at 30°C with shaking for 24 h, then resazurin was added to each well, and a change in color of resazurin in the 96-well assay plate was determined by visualization. From the primary screening of the 14 compounds, two flavonoid candidates, 5,6,7,8,3’,4’,5’-heptamethoxyflavone (**4**) and 3,5,7,8,3’,4’,5’-heptamethoxyflavone (**9**), were found to be positive for hCAII inhibition (Table 1).

The minimal effective concentration (MEC) of these two flavonoids (**4** and **9**) for inhibition of hCAII was evaluated testing two-fold serial dilutions in the developed yeast-based assay. Both compounds **4** and **9** showed potential hCAII inhibition activity at these lower concentrations with MEC values of 10.8 and 21.5 μM, respectively, (Table 2; Fig. 2B). The growth of the yeast indicator cell when also determined as the OD_660_ (Fig. 2A) was consistent with the observed visual color-change (Fig. 2B).

Several recent studies have shown that phenolic or flavonoid molecules have CA inhibitor activities [37, 38]. For example, the effect of some flavonoids on the inhibition of hCAI and hCAII activities in vitro was determined in terms of the CO_2_-hydratase activity and esterase activity, where the tested flavonoids effectively inhibited both hCAI and hCAII [37]. This suggested that flavonoids may be effective CA inhibitors, in spite of unknown specificity. However, eliminating compounds with the worst ADMET (Absorption, Distribution, Metabolism, Excretion and Toxicity) properties is important in the early drug discovery process [45], and using only the in vitro assays could not provide the ADMET properties of the hit compounds.

Since this assay is based on the growth inhibition of the yeast indicator cells, cytotoxic compounds would also be selected in the screening system. However, these compounds can be easily eliminated by examining the growth of the assay cells treated with the test compound under a high CO_2_ condition, where the growth of the yeast indicator cells does not depend on the induced hCAII activity [46]. The candidate compounds **4** and **9**, were determined for their cytotoxic effect at the tested concentrations by incubation of the assay plate at 30°C with shaking under a high CO_2_ condition. No cytotoxic effect of either compound **4** or **9** was observed even at the highest concentration tested (Figs. 2C and 2D), where no minimal toxic concentration (MTC) could be detected even at a concentration as high as 170 μM.

### Esterase Activity

**Fig. 3:**
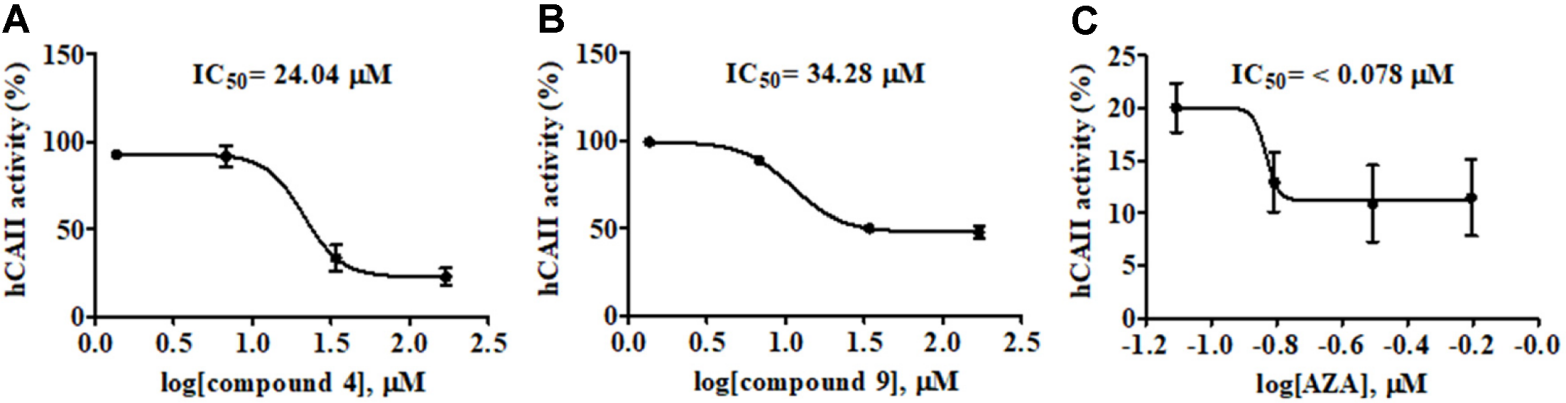
The IC_50_ determination of the hCAII inhibitors: (A) compound 4, (B) compound 9, and (C) AZA. The hCAII was incubated with various concentrations of inhibitor for 2 h and the formation of 4-NP from 4-NPA was followed at OD_405_ nm. The IC_50_ values were calculated from triplicate data using GraphPad Prism, version 5.01 by a nonlinear regression. Data are shown as the mean ± 1SD derived from three independent experiments. Statistical significance was analyzed under a dose-response inhibition (log[inhibitor] vs. response-variable slope) model.

The activity of CAs can be screened in terms of the hydrolysis of the ester 4-NPA to release 4-NP where the absorbance of 4-NP is monitored at 405 nm [47]. To confirm that compounds **4** and **9** contain anti-hCAII activity, their in vitro inhibition activity of the esterase activity against 4-NPA was ascertained, and revealed an esterase inhibitory activity with IC_50_ values of 24.0 and 34.3 μM for compounds **4** and **9**, respectively, (Table 2; Fig. 3). The relatively close values between the MEC from the yeast-based assay and the IC_50_ from the biochemical assay of compounds **4** and **9** reflected their properties on cell permeability, solubility and stability inside the cells (Table 2).

### Evaluation of the Potential Inhibition Mechanism by In Silico Molecular Docking

**Fig. 4:**
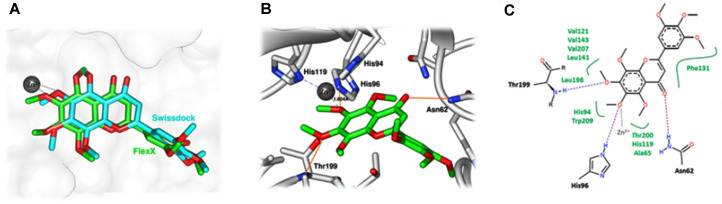
In silico molecular docking of compound 4 with hCAII. (**A**) Comparison of the docked structures of compound **4** in the active site of hCAII obtained from the FlexX (green stick model) and SwissDock (blue stick model) molecular docking approaches. (**B**) Binding interactions of compound **4** with the hCAII amino acids as well as the Zn^2+^ ion, as obtained from FlexX, where the Zn^2+^ coordination and intermolecular hydrogen bond are shown by a dashed line and orange solid line, respectively. (**C**) The 2D interaction diagram of compound **4** with hCAII, where the Zn^2+^ coordination and hydrogen bonds are presented by black and red dashed lines, respectively, while the contact amino acids contributing to compound **4** binding via van der Waals interactions are shown in green text.

**Fig. 5:**
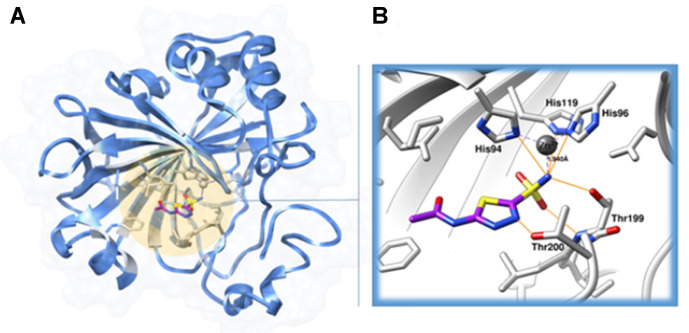
In silico molecular docking of AZA with hCAII. (**A**) The co-crystal structure of the AZA/hCAII complex, 3HS4.pdb, where in panel (**B**) hydrogen bonds formed between AZA (magenta stick model) and the catalytic triad His94, His96 and His119 as well as the gate-keeping residue Thr199 are depicted by orange lines, while dashed lines represent the Zn^2+^ coordination.

To investigate the potential inhibition mechanism of compound **4**, selected as it had the highest biological activity towards hCAII, in silico molecular docking was performed using FlexX and SwissDock. Both in silico approaches revealed a similar conformation of compound **4** in the CAII active site (Fig. 4A), coordinating with the Zn^2+^ ion through its methoxy oxygen at a distance of 1.60 Å (FlexX) or 2.29 Å (SwissDock), which is in the range reported previously for bis(3,4-dimethoxyphenyl) methane [48]. Hereafter, the obtained results from the FlexX simulation (Figs. 4B and 4C) were chosen for further discussion in terms of the inhibitor-target interactions.

Besides the formation of the metal/ligand complex, compound **4** was well stabilized by the imidazole ring of His96 (one of the catalytic triad), the amide group of Asn62 and the peptide nitrogen of Thr199 (the gate-keeping residue) by the formation of three hydrogen bonds. The other two catalytic triad residues (His94 and His11) and the non-polar residues (Ala65, Val121, Phe131, Leu141, Val143, Leu198, Thr200, Val207, and Trp209) at the binding area of hCAII likely contributed via van der Waals interactions. These key residues have previously been found to play an important role in stabilizing several CA inhibitors [49-53]. The interaction energy of the compound **4**/CAII complex was -13.36 kcal/mol. The overview of how compound **4** might inhibit hCAII at a molecular level could be useful for further anti-hCAII design and development based on this potent flavonoid compound.

In conclusion, this study utilized our previously developed yeast-based assay [18] to screen for compounds with anti-hCAII activity. Among 14 natural compounds (flavonoids and coumarin compounds) isolated from *M. paniculata*, two flavonoid compounds, compounds **4** and **9**, were found to be positive with MEC values in the yeast assay of 10.8 and 21.5 μM, respectively, and MTC values higher than 170 μM (the highest concentration tested). The results of the in vitro esterase inhibition activity were consistent with those obtained from the yeast-based assay. Molecular docking of compound 4 with hCAII revealed that it coordinates with Zn^2+^ in the active site of CA through hydrogen bonding, electrostatic and van der Waals interactions in a similar manner to AZA, a known CA inhibitor.

The flavonoid compounds **4** and **9** from *M. paniculata* were found to be potent hCAII inhibitors with no cytotoxicity (at the highest concentration tested), good solubility, permeability and stability. Although their inhibitory activities were not as potent as known CAI (AZA), they are novel natural inhibitors of hCAII and so could potentially serve as non-sulfonamide based lead compounds for further drug development for glaucoma treatment.

## Figures and Tables

**Table 1:** Primary screening of compounds isolated from *M. paniculata* for anti-hCAII activity using the yeast-based assay.

Compound	Reference^a^	hCAII inhibition^b^
Minumicrolin **(1)**	[54]	-
2,6,2′,6′-Tetramethoxy-4,4′-bis(1,2-trans-2,3-epoxy-1-hydroxypropyl)biphenyl **(2)**	[55]	-
Murrangatin acetate **(3)**	[56]	-
5,6,7,8,3′,4′,5′-heptamethoxyflavone **(4)**	[57]	+
Phebalosin **(5)**	[58]	-
3,5,6,7, 3′,4′,5′-heptamethoxyflavone **(6)**	[59]	-
Auraptenol **(7)**	[22]	-
-(-) Murrangatin **(8)**	[60]	-
3,5,7,8,3′,4′,5′-Heptamethoxyflavone **(9)**	[59]	+
Murralongin **(10)**	[56]	-
3,5,7,3′,4′, 5′-Hexamethoxyflavone **(11)**	[61]	-
Muralatin K **(12)**	[62]	-
Murracarpin **(13)**	[60]	-
Omphalocarpin **(14)**	[63]	-
Acetazolamide (AZA) (as a positive control)		+

^a^Reference used for NMR data identification of the compound

^b^Inhibition of hCAII in the yeast AS03(pGAL1.1-hCAII) cell assay: + (inhibition); - (no inhibition)

**Table 2:** Efficacy of compounds 4 and 9 in the in vivo yeast-based assay and in vitro hCAII activity inhibition.

Compound	Yeast-based assay (μM)		In vitro assay (μM)

	MEC	MTC	IC_50_
4	10.8	> 170	24.04
9	21.5	> 170	34.28
Acetazolamide (AZA) (a positive control)	0.31	> 0.63	< 0.078
